# Rethinking clinical trials for medical AI with dynamic deployments of adaptive systems

**DOI:** 10.1038/s41746-025-01674-3

**Published:** 2025-05-06

**Authors:** Jacob T. Rosenthal, Ashley Beecy, Mert R. Sabuncu

**Affiliations:** 1 Tri-Institutional MD-PhD program of Weill Cornell/Rockefeller/Sloan Kettering, New York, NY USA; 2 Department of Radiology, Weill Cornell Medicine, New York, NY USA; 3 Division of Cardiology, Department of Medicine, Weill Cornell Medicine and NewYork-Presbyterian, New York, NY USA; 4 School of Electrical and Computer Engineering, Cornell Tech and Cornell University, New York, NY USA

**Keywords:** Machine learning, Health care

## Abstract

There is a growing recognition of the need for clinical trials to safely and effectively deploy artificial intelligence (AI) in clinical settings. We introduce dynamic deployment as a framework for AI clinical trials tailored for the dynamic nature of large language models, making possible complex medical AI systems which continuously learn and adapt in situ from new data and interactions with users while enabling continuous real-time monitoring and clinical validation.

## Introduction

Artificial intelligence (AI) in medicine has been riding atop a wave of inflated expectations and hype for at least a decade^1^, with the pace of research continuing to accelerate in recent years driven by the advent of large language models (LLMs)^2,3^. Scientists and clinicians have sought to take advantage of these technological advancements by applying LLMs to a wide array of areas in the healthcare system, ranging from estimating causal treatment effects of medications from online forum posts^4^, to aiding in writing research articles^5^, and automating administrative tasks such as insurance prior authorization paperwork^6^. Among many in the healthcare field, there is a general consensus that AI in healthcare is “the future”^7^.

The lifecycle of an AI model can be conceptualized in stages, beginning with initial problem identification, then proceeding to a design phase, followed by model development in the research setting, silent deployment, then deployment to “production” setting in the real-world, and finally a post-deployment phase of monitoring and making changes or removing from production as needed^8–11^. This has similarities to models of the software development lifecycle^12^.

In the medical context, there is a growing recognition of the need for deployment to happen through clinical trials, so as to protect participants and rigorously ensure the safety and efficacy of models^13^. Yet while there have been some notable examples of prospective clinical trials of LLM tools, such as for aiding nurses with receptionist tasks^14^ and drafting responses to patient messages^15^, overall only a very small fraction of models ever makes it out of the research phase to be deployed in the real-world setting. A systematic review in 2022 found only 41 randomized trials of machine learning interventions worldwide^16^; by 2024, this number had increased to a total of only 86^17^. A 2023 analysis of insurance claims found a total of only 16 medical AI procedures with billing codes^18^. Overall, the medical system has failed to keep up with the pace of recent developments in AI – this disconnect, known by various terms such as “implementation gap”^19^ and “AI chasm”^20^, means that the vast majority of research advances in medical AI never actually directly benefit patients or clinicians. The causes of the implementation gap are multifactorial and include not only technical and logistical barriers, but also sociocultural, ethical, economic, and regulatory factors^21–26^. Bridging the implementation gap is one of the largest challenges currently facing the field of medical AI.

In this piece, we describe the currently predominant approach to medical AI deployment, which is based on a linear, model-centric understanding of AI. We then identify several shortcomings of this paradigm when applied to LLM-based systems and propose an alternative way of conceptualizing AI in medicine based on continual processes of model updating, real-world evidence generation, and safety monitoring, which we call dynamic deployment. We chart a path towards making such dynamic deployments a reality, drawing on well-established methods of adaptive clinical trials as well as more recent technical advances and developments in regulatory science of medical AI.

## Linear model of AI deployment

**Fig. 1:**
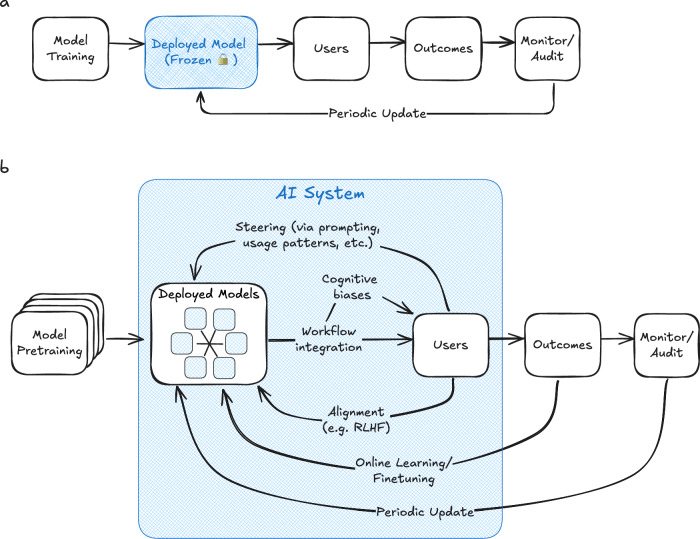
Depiction of the linear and dynamic models of AI deployment. **a** In the linear model of AI deployment, a model is first trained in the research/development setting, then deployed to the real-world setting with its parameters frozen. Model weights may be updated periodically following post-deployment monitoring and auditing. **b** In the dynamic framework, models are first pre-trained in the research/development setting. They remain dynamic when deployed, and mechanisms are in place to enable continuous updating in response to feedback signals from their deployment environments (arrows). Multiple AI models may be simultaneously deployed and interacting. All elements inside the blue box are considered part of the complex AI system, including the AI models, the users, the workflow integrations and interfaces by which they interact, and the feedback and update mechanisms.

Where AI models have been successfully deployed in healthcare, they typically follow a pattern which we refer to as the linear model of AI deployment (Fig. 1a). First, a model is developed in the research domain, most often by training on retrospective data. The model is then assessed, and its performance characteristics evaluated. When the decision is made to move the model from research into deployment, it is frozen: all the model’s parameters are locked and remain static for as long as it is deployed. Although it could be updated periodically in response to new data or performance degradations identified through post-deployment monitoring and auditing, there are few examples of this happening in practice.

In the linear framework, the focus is on a particular AI model. More specifically, it is a particular instance of the given model defined by its set of parameters. The linear model is intuitive and closely mirrors the process by which other technologies are brought into clinical practice. However, the linear model of AI deployment is a poor fit for modern LLM systems, for three principal reasons which we outline below.

1. AI is an adaptive technology
  AI systems have an important difference from other technologies in medicine: they are adaptive. In fact, one of the most important attributes of modern LLMs with billions of parameters is their flexibility. Model weights do not necessarily need to remain fixed throughout the lifespan of the model’s deployment, and can be periodically finetuned or updated as batches of new data come in. Methods such as reinforcement learning from human feedback (RLHF)^27^ and direct preference optimization (DPO)^28^ also allow LLMs to learn directly from their users in order to be better aligned with user preferences, and recent work has extended these approaches to the “online learning” setting, allowing for continuous updating of deployed models^29,30^. The behavior of LLMs can also be substantially changed during deployment through interactions with users, without updating any of the model parameters: for example, in-context learning allows LLMs to learn from new training data presented in their prompts^31^, and chain-of-thought prompting enables LLMs to more effectively reason through complex problems^32^. For all these reasons, the line between model development and model deployment is becoming increasingly blurred. Consequently, it is unclear how the linear model of AI deployment would handle such interactive and dynamic features of emerging AI systems. By relying on the underlying assumption that learning occurs only in discrete phases, the linear model struggles to encompass many of the most promising avenues of modern AI advances, especially with regards to the interactive and dynamic nature of LLMs.
2. AI functions as part of a complex system
  Secondly, the linear model does not sufficiently account for the complex systems in which AI models are employed. The outputs of the model itself are of course crucial, but are only one part of the system. Other factors beyond the model parameters also drive outcomes. For example, choices related to user interface design can shape interactions between humans and AI models, introducing new cognitive biases into clinical decision-making^33–36^. Even when clinicians are given access to LLM systems with super-human abilities, human users will not necessarily be able to effectively take advantage of the full potential of these tools without specialized training^37^. The behaviors of interactive AI systems, such as chatbots, also depend integrally on the behavioral patterns and values of the particular population of users^38^. Thus, even when model weights are frozen, the system is not static. By adopting a model-centric, parameter-centric view of AI, the linear model fails to adequately account for the numerous other factors contributing to meaningful outcomes in the real world.
3. Health systems of the future will have many AI models operating at once.
  Finally, the linear model of AI relies on the premise of isolating a single model for testing. This is reasonable today, as there are so few AI models in the wild. However, it potentially poses a major challenge for scaling up the extent of AI integration. In the near future, there may be orders of magnitude more models deployed in various contexts throughout the medical system. Users may interact with many different models during their routine workflows, and models could interact with each other and be interdependent in complex ways. This is exemplified by the emerging paradigm of multi-agent AI systems, whereby tasks are completed by a cohort of individual LLM-based agents, orchestrated by other “supervisory” models^39–41^. In such scenarios, AI clinical trial designs which seek to evaluate the behavior of a specific model in isolation would be impractical.

## Dynamic systems model of AI deployment

To overcome these challenges, we propose an alternative framework for clinical trials and deployment of LLMs, which we call dynamic deployment (Fig. 1b). In a nutshell, the dynamic deployment model is distinguished from the linear deployment model in two key ways: 1) by embracing a systems-level understanding of medical AI, and 2) by explicitly accounting for the fact that such systems are dynamic and constantly changing. In this section we describe the framework and discuss how it can be applied in the real world through adaptive clinical trials.

The first principle is a systems-level approach to medical AI. In this model, the AI system is conceptualized as a complex system with multiple interconnected moving parts. The AI model itself is at the core and functions the same as in the linear model: taking input data and producing outputs according to its internal parameters. What sets apart this approach, however, is that other elements in the AI system are also explicitly included as parts of the intervention. This includes the population of users, each guided by their own set of values and behavioral patterns; the workflow integration and user interface by which users interact with models; and other automated elements, such as the data generation or processing pipelines and the update mechanisms for online learning. Each individual component contributes to the overall behavior of the system, although disentangling the exact contribution from each element might not be feasible. However, the systems-level view says that it is not actually necessary to measure these complex intra-system relationships. What matters is the behavior of the system as a whole, as measured by metrics that are meaningful in the real-world, such as patient outcomes^42^. For example, gradual degradation of performance metrics over time is a clear indicator that the system as a whole is not functioning well, even though it may be difficult or impossible to isolate the effects of AI model degradation from other sources of variation such as natural fluctuation in patient or user populations. A systems-level approach aims to use feedback loops to learn from these performance changes over time, regardless of their root causes. By shifting focus to a systems-level conceptualization of medical AI, we will be able to better measure things which actually matter.

The second principle informing the design of dynamic medical AI deployments is the recognition that they are systems which change over time. AI models still undergo an initial research and development phase before being deployed, however this is understood to be “pretraining,” i.e. the start of training rather than the end. Instead of models being frozen, they are allowed to continue to evolve in response to feedback signals during deployment. These can occur by mechanisms such as online learning or finetuning with new data, alignment with user preferences via RLHF or DPO, or more subtle causes such as drift in user populations altering system behavior due to differences in usage patterns. To provide concrete examples, we list several concrete examples of feedback signals in Table 1 and mechanisms of adaptation in response to these signals in Table 2. Rather than trying to freeze the system and measure its performance at discrete snapshots in time, the dynamic approach relies on feedback loops allowing for both continuous iteration and continuous evaluation. Discrete, post-deployment updates and audits are augmented by their continuous analogs, allowing for AI systems to continually update in response to new data.

**Table 1:** Selected examples of sources of AI system performance feedback which can be monitored and used to improve performance via feedback loops

Feedback signal
New patient data
Patient outcome metrics derived from EHR data (e.g. readmission rate within 30 days)
Workflow metrics (e.g. average physician time spent per note)
Human expert review of AI outputs
User feedback (e.g. solicited through surveys or obtained indirectly through analysis of usage patterns)
Patient satisfaction surveys
Experiments (e.g. A/B testing)
Periodic audits

**Table 2:** Selected examples of mechanisms by which AI systems behavior can be modified in response to feedback signals

System adaptation mechanism
Modify model weights directly (e.g. finetuning, online learning)
Change underlying LLM (e.g. upgrade to a new version, switch to a new vendor)
Modify user interfaces
Develop training modules for users (e.g., best practices for prompting LLMs)
Nudge user behavior directly (e.g. AI agent gives real-time feedback to prompt a human user to rephrase a sentence)
Automatically optimize LLM system prompts^59,60^
Add or remove guardrails to constrain LLM outputs
Add new AI agents to perform certain tasks, or give agents access to new tools

To this extent, deployment itself can be thought of as another phase of the model-generation process whereby the model learns directly from its intended users and from new data as it comes in. In this sense, the linear notion of “train → deploy → monitor” is replaced by a system in which all three processes are happening at once. Treating medical AI systems as dynamic is more faithful to their real-world behavior and allows for intelligent systems which take maximal advantage of all available data and learn from every participant.

We note that if all the feedback flows (i.e., online learning, alignment, prompting, steering, etc.) are removed from the dynamic model, the result is a linear model. Therefore, the linear model is a special case of the dynamic model. The dynamic model simply formalizes and makes explicit the routes of information flow and system evolution which are implicitly present in all linear AI deployment systems.

## Adaptive clinical trials for medical LLM deployment

### Deployment and clinical validation

One of the most urgent challenges for medical AI is clinical validation. Deep learning models, especially LLMs, are largely empirical with few theoretical performance guarantees, meaning that our ability to characterize their real-world behavior in the research setting is limited. Retrospective analyses are often used to estimate the likely behavior and impact of AI models when deployed, but these are imperfect proxy measures and reliance on them can ultimately make AI systems more risky and potentially lead to unforeseen behavior^43^. Recent work has stressed the importance of real-world deployment for evaluating real-world model effectiveness^44^ and highlighted how model performance metrics assessed during training and development may change when deployed in the real world^45–47^.

However, a recent study of the 521 medical AI devices approved by the FDA found that more than 40% lacked any such clinical validation data^48^. Generative AI tools available to the general public are also being widely used in clinical settings, despite the fact that presumably none of them were validated or officially approved for medical use: a recent survey of 1000 doctors in the United Kingdom revealed that 20% of respondents reported using generative AI tools in their practice^49^. Using AI tools without clinical validation comes at the cost of increased risk of unforeseen consequences leading to negative outcomes and decreased trust among patients, clinicians, and the public.

Dynamic deployments help address this problem because continual performance monitoring is baked into the system design. Not only is deployment the only way to deliver the promises of AI in medicine to make tangible impacts on real patients and clinicians, but it is also the only way to directly study the behavior of AI models in situ. In addition to providing supervisory signal for online learning and other feedback mechanisms, these performance metrics can be used for real-time monitoring and oversight. By including performance assessment as a core principle in designing AI systems, each deployment can be viewed as a sort of local clinical trial; such recurring local validations may actually be better suited for modern AI systems than the alternative paradigm of external validation which multi-site clinical trials are based on^50^.

### Existing precedent

At first blush, the proposed shift towards dynamic AI systems may seem to make clinical deployment even more difficult than it already is, possibly even widening the implementation gap. However, this need not necessarily be the case. In this section, we chart the path towards making dynamic medical AI a reality.

While AI is a new technology, forms of dynamic deployment have long been used in early-stage clinical trials to navigate the high degree of uncertainty in benefit/harm profile often seen in phase I clinical trials. For example, adaptive continual reassessment uses a Bayesian framework to learn from new data as it comes in and continually update the algorithm responsible for assigning patients to trial arms^51^. First developed more than 30 years ago, such adaptive trial designs are still being used today^52^. Not only does this approach satisfy an ethical concern by ensuring that no patient is given a treatment which is known to be inferior, but it also appeals to statistical efficiency by utilizing all possible data gleaned from previous trial participants^51^. Conceptually, this can be viewed as a form of dynamic deployment, where the AI model is a Bayesian model as opposed to an LLM, and online learning is used to continuously optimize the model parameters in response to patient outcomes. Guidelines for protocol design and reporting of clinical trials involving AI considered such continuously learning trial designs as “of interest” but intentionally excluded them as still too “early in development”^53,54^. However, because such adaptive trial designs are in fact already well-established and are already relied upon for making policy and treatment decisions, they could represent a promising blueprint for pursuing dynamic deployments of medical AI systems without the need to invent entirely new regulatory mechanisms.

### Challenges

Practical challenges remain which must be addressed to enable widespread deployment of dynamic medical AI systems. First, building and maintaining infrastructure for feedback loops will require investment on the part of hospitals and health systems. Patient outcome metrics, although most important, may also be the most difficult to collect, necessitating patient follow-up, data integration and automated abstraction from health records, and such real-world evidence has known limitations^55^. As AI usage expands, costs for computational infrastructure and AI services could also quickly grow; care must be taken to ensure that dynamic LLM deployments are cost-effective for institutions^56,57^. Further, as many of the leading LLMs are currently proprietary closed-source models accessed through vendor services which do not allow modifications to be made to the underlying parameters, the options for finetuning models may be constrained. Data privacy and cybersecurity concerns, while not unique to AI, will continue to be of critical importance. Finally, institutions may be hesitant to try new models such as dynamic deployment given the rapidly evolving regulatory and medicolegal landscape of medical AI. Developing sustainable models of AI governance and quality oversight will be an essential task for regulatory bodies and local leadership, enabling medical AI integration while ensuring appropriate balance between oversight and innovation. Recent FDA guidance on predetermined change control plans^58^ is an important step in this direction.

## Conclusion: looking forward

The current regime of linear AI deployment has largely failed to keep up with the pace of technological development and is a poor fit for the emerging paradigm of interactive, adaptive, multi-agent AI systems. We propose dynamic deployments as an alternative framework for medical AI deployments which are continually learning and adapting in response to new data, shifting focus beyond individual AI models towards a system-level perspective. Dynamic deployments can be used in the context of intervention arms in AI clinical trials to facilitate comparison with control groups and estimation of the causal effect of AI system implementation. They could also be used in the absence of control groups to deploy AI systems which learn and adapt over time.

Not all use cases will be amenable to such dynamic systems. Tasks with a highly predictable structure, such as image-based diagnostics, are less likely to benefit than unstructured tasks such as note writing. Additionally, in high-risk applications such as surgical robotics, or for fully autonomous systems with no humans in the loop, the benefits of continual learning might be outweighed by the risks. Careful oversight is necessary to govern appropriate use of dynamic AI systems, and these decisions will be highly context-dependent. For those cases where dynamic deployment is a good fit, continually learning AI systems present a promising path towards maximizing positive impact.

In the future of medicine, AI will likely be integrated in innumerable ways throughout the healthcare system. Hospitals will take advantage of intelligent, adaptive workflows, and healthcare will expand its reach to be more accessible than ever before. The current state of AI in medicine is analogous to the early days of the internet in the late 1990s: the core technologies are ready, but the field has not yet developed a mature, robust ecosystem to make it broadly useful beyond a core group of early adopters and enthusiasts. The next generation of medical AI will similarly be ushered in when we step back from individual models and instead focus on the larger picture of adaptive systems and networks, building upon the core principles of safety, real-world evidence, and regulatory oversight.

## Data Availability

No datasets were generated or analysed during the current study.
